# Clusterin is enriched in plaque‐associated astrocytes and localizes to synapses in Alzheimer's disease

**DOI:** 10.1002/alz.71885

**Published:** 2026-10-06

**Authors:** Robert A. J. Byrne, Aurora Veteleanu, Hannah L. Cramp, Olivia G. Bacon, Kirsten L. Baillie, Matthew Bright, Rebekah S. Cooke, Gareth D. Fenn, Mariana Flores Castelán, Kok Yung Lee, Wioleta M. Zelek, B. Paul Morgan, Nikoleta Daskoulidou

**Affiliations:** ^1^ UK Dementia Research Institute, School of Medicine Cardiff University Cardiff Wales UK; ^2^ Hodge Centre for Translational Neuroscience, School of Medicine Cardiff University Cardiff Wales UK

**Keywords:** Alzheimer's disease, astrocytes, clusterin, complement, immunoassay, monoclonal antibody, synapses

## Abstract

**INTRODUCTION:**

The clusterin gene (*CLU*) is a top genome‐wide association studies (GWAS) risk locus for Alzheimer's disease (AD) and elevated clusterin levels have been reported in AD biofluids and brain tissue. Despite growing interest in clusterin as a biomarker and therapeutic target, its cellular and subcellular localization in the human brain remains incompletely understood, in part due to a lack of well‐characterized reagents.

**METHODS:**

We generated and validated monoclonal antibodies (mAbs) that distinguish secreted and intracellular clusterin, and applied these tools to human biofluids, brain tissue, and induced pluripotent stem cell (iPSC) ‐derived astrocytes.

**RESULTS:**

The mAbs enabled sensitive detection and quantification of clusterin in plasma, serum, cerebrospinal fluid, and brain tissue homogenates. Immunostaining demonstrated that clusterin is associated with amyloid plaques in AD brain and localizes to synapses in both healthy and AD tissue; the mAb detecting intracellular clusterin demonstrated specific expression in astrocytes, confirmed using astrocytoma lines and iPSC‐derived astrocytes. Quantitative analysis demonstrated increased clusterin signal in plaque‐associated astrocytes, particularly those surrounding larger plaques.

**DISCUSSION:**

The findings provide better understanding of clusterin expression in the healthy and AD brain, informing roles of clusterin in brain homeostasis and AD pathogenesis.

## Highlights

- Novel antibodies distinguish secreted and intracellular clusterin isoforms.
- Clusterin is expressed intracellularly in astrocytes in healthy and Alzheimer's brain.
- Clusterin expression is increased in periplaque astrocytes in Alzheimer's brain.
- Secreted clusterin labels amyloid plaques in Alzheimer's brain and synapses in healthy and Alzheimer's brain.

## 1 BACKGROUND

Alzheimer's disease (AD) is a debilitating neurodegenerative disorder characterized clinically by memory loss, attention deficits, diminished executive function, and pathologically by accumulation of toxic protein aggregates, notably extracellular amyloid beta (Aβ) plaques and intracellular hyperphosphorylated tau tangles.^1^ In the past two decades, genome‐wide association studies (GWAS) have shown that late onset AD is a highly polygenic disorder, with over 70 genome‐wide significant risk loci identified to date.^2^ The first adequately powered AD GWAS implicated the clusterin gene (*CLU)*, encoding clusterin (also known as apolipoprotein J), as an important AD risk gene,^3^, ^4^ a finding that has been robustly replicated in subsequent GWAS.^2^, ^5^, ^6^ Clusterin is a 80 kDa heterodimeric glycoprotein ubiquitously expressed in biofluids that performs numerous physiological functions, including cholesterol transport,^7^ regulation of the complement system,^8^, ^9^ and molecular chaperone roles.^10^ In addition to this secreted form present in biofluids, intracellular cytoplasmic and mitochondrial forms exist with different structural properties and poorly characterized functions. Intracellular clusterin isoforms arise from alternative splicing, with cytoplasmic and mitochondrial *CLU* transcripts both omitting exon 2 but initiating translation at different residues in exon 3, resulting in non‐glycosylated 50 and 45 kDa proteins, respectively.^11^, ^12^, ^13^ Furthermore, *CLU* genotype appears to impact expression of specific clusterin isoforms. The major allele of rs11136000, the original *CLU* index risk variant identified in AD GWAS,^3^, ^4^ is associated with reduced expression of both secreted and intracellular clusterin in brain tissue; in contrast, the protective minor allele is associated with elevated intracellular clusterin expression, but not secreted clusterin.^14^ Hence, risk variants in *CLU* may confer AD susceptibility by altering the ratio of clusterin isoforms in the brain.

Clusterin interaction with Aβ is well documented, with clusterin shown to localize to Aβ plaques,^15^, ^16^ inhibit Aβ oligomerization and aggregation in vitro,^17^, ^18^, ^19^ and promote Aβ clearance through receptor‐mediated endocytosis.^20^, ^21^ However, the consequences of these interactions in vivo and how they impact disease risk are poorly understood. Experimental studies have reported both protective and pathogenic roles for clusterin in AD; manipulation of clusterin expression influences amyloid deposition, cognition, and disease progression in multiple AD mouse models. In the APP/PS1 AD mouse model (expressing a chimeric mouse/human mutant amyloid precursor protein [APP695Swe] and a mutant human presenilin‐1), *Clu* overexpression reduced Aβ load while heterozygous *Clu* knockout (APP/PS1 *Clu^+/−^*) caused elevated Aβ load.^22^ Conversely, *Clu* double knockout (APP/PS1 *Clu* ^−/−^) mice displayed reduced parenchymal Aβ load, with plaques accumulating near blood vessels.^23^ In PDAPP mice (overexpressing human amyloid precursor protein APP717V→F), *Clu* deficiency reduced fibrillar Aβ deposits but did not affect total Aβ levels, while combining *Clu* and apolipoprotein E (*APOE)* deficiencies in PDAPP mice resulted in earlier disease onset and elevated Aβ deposition.^24^, ^25^ Furthermore, in 5xFAD mice (overexpressing mutant human APP with three familial AD mutations and human PS1 with two familial AD mutations), *Clu* deficiency resulted in impaired cognition and reduced Aβ levels at 5 months.^26^ Similarly, studies investigating interactions between clusterin and tau yielded conflicting findings, with both neuroprotective and pathogenic effects reported. Clusterin inhibited tau fibril formation in vitro and elevated tau pathology and anxiety‐like behavior were observed in mutant tau‐overexpressing *Clu^−/−^* mice, indicating a neuroprotective role.^27^ In contrast, others reported that clusterin increased seeding of tau aggregates between neurons, suggesting a pathogenic role.^28^ Together, these findings illustrate the complex role for clusterin in clearance of toxic protein aggregates and AD pathogenesis.

Numerous studies have investigated clusterin as a candidate AD biomarker; elevated clusterin levels have been reported in AD plasma,^29^, ^30^, ^31^ cerebrospinal fluid (CSF),^32^ and brain tissue.^33^, ^34^ Elevated clusterin levels in CSF and plasma were associated with accelerated disease progression^30^, ^32^ and AD risk; plasma clusterin levels positively correlated with AD polygenic score,^35^ although AD‐associated polymorphisms in *CLU* had no impact on clusterin protein and mRNA levels.^31^, ^33^, ^35^, ^36^ Despite these observations, the mechanisms linking clusterin to AD remain poorly understood. In particular, it remains unclear whether disease‐associated changes reflect extracellular clusterin, intracellular clusterin, or both, and how these distinct forms are distributed within the human brain.

Progress in understanding clusterin biology has been hindered by a lack of well‐characterized reagents capable of distinguishing between secreted and intracellular forms of the protein. Commercial mAbs are often poorly validated and their specificity for different clusterin isoforms is rarely defined, limiting interpretation of localization and biomarker studies. To address this, we generated and characterized a panel of anti‐clusterin mAbs that differentiate secreted and intracellular clusterin and used these reagents to detect secreted and intracellular clusterin in tissues and cells. The findings shed new light on clusterin expression and location in healthy and AD brain.

## 2 MATERIALS AND METHODS

### 2.1 Chemicals

Reagents and chemicals were of analytical grade and were purchased from Fisher Scientific unless stated otherwise. Phosphate‐buffered saline (PBS) comprised 137 mM NaCl, 2.7 mM KCl, 10 mM Na_2_HPO_4_, 1.8 mM KH_2_PO_4_, pH 7.4. HEPES‐buffered saline (HBS) comprised 10 mM HEPES, 150 mM NaCl, 135 µM CaCl_2_, 1 mM MgCl_2_, pH 7.4.

**RESEARCH IN CONTEXT**

1. **Systematic review**: The authors reviewed the literature using PubMed and Google Scholar searches focused on clusterin (CLU), Alzheimer's disease (AD), astrocytes, synapses, amyloid plaques, and complement. Previous studies established CLU as a major genetic risk factor for AD and demonstrated associations between CLU and amyloid beta aggregation, plaque deposition, and disease progression. CLU has also been proposed as a biomarker in AD, although findings across studies have been inconsistent. Despite extensive interest in CLU biology, the cellular and subcellular distribution in the human AD brain remains incompletely understood.
2. **Interpretation**: Using newly validated monoclonal antibodies capable of distinguishing secreted and intracellular CLU, we explored CLU distribution in healthy and AD brain. Intracellular CLU was specifically detected in astrocytes; expression was significantly increased in astrocytes surrounding amyloid plaques, suggesting a localized response to amyloid pathology. Secreted CLU localized to plaques in AD brain and to synapses in both control and AD brain, the latter suggesting a role in synaptic homeostasis and neurodegeneration. These findings provide new insight into expression and localization of CLU isoforms in AD and healthy brain, essential to understand its string genetic association with AD risk.
3. **Future directions**: Future work should determine how the intracellular and secreted isoforms of clusterin contribute to AD pathogenesis, including potential roles in complement regulation, synaptic maintenance, and plaque‐associated neuroinflammation. The capacity to distinguish secreted from intracellular CLU opens new directions for mechanistic and biomarker studies to reveal how CLU genetic risk impacts disease progression.

### 2.2 Cell culture

SP2/0‐Ag14 mouse myeloma cells (European Collection of Animal Cell Cultures) were cultured in RPMI 1640 medium with 2 mM glutamine, 2% (w/v) penicillin/streptomycin, 1 mM sodium pyruvate, and 15% (v/v) fetal bovine serum (FBS; Gibco) at 37°C with 5% CO_2_. 1321N1 astrocytoma cells (kindly donated by Professor Kathy Triantafilou, Cardiff University) were cultured in Dulbecco's Modified Eagle Medium (DMEM) with 2 mM glutamine, 2% (w/v) penicillin/streptomycin, 1 mM sodium pyruvate, and 10% (v/v) FBS at 37°C with 5% CO_2_.[Fig alz71885-fig-0001], [Fig alz71885-fig-0002], [Fig alz71885-fig-0003], [Fig alz71885-fig-0004], [Fig alz71885-fig-0005], [Fig alz71885-fig-0006], [Fig alz71885-fig-0007]

Induced pluripotent stem cell (iPSC) lines KOLF2.c1 (Wellcome Trust Sanger Institute), A1, B1, F1, and H1^37^, ^38^ were cultured on plates coated with Geltrex (1:100; Thermo Fisher Scientific, A1413201) with mTeSR Plus medium (STEMCELL Technologies) at 37°C in 5% CO_2_. Differentiation of iPSCs into astrocytes was performed according to published methods.^37^, ^39^ Conditioned medium (2 ml) was collected on day 7 of terminal differentiation and subjected to trichloroacetic acid (TCA) precipitation by adding TCA to 10% (w/v), incubating on ice (30 minutes), centrifuging at 10,000 × *g* (10 minutes at 4°C), acetone‐washing the pellet, air‐drying and re‐dissolving in 100 µl PBS. Total protein in post‐TCA precipitation conditioned medium was measured via bicinchoninic acid (BCA) assay (Thermo Fisher Scientific, #23235).

### 2.3 Human tissue

Cryopreserved *post‐mortem* brain tissue from one AD case (Braak VI) and five controls was provided by the Edinburgh Brain Bank (Table S1). Preliminary immunohistochemistry was performed on the AD sample; synaptoneurosome isolation was performed on the control samples according to published methods^40^ and validated during a previous study.^41^ Use of human tissue for *post‐mortem* studies was reviewed and approved by the Edinburgh Brain Bank Ethics Committee and the Medical Research Ethics Committee (the Academic and Clinical Central Office for Research and Development, a joint office of the University of Edinburgh and National Health Service (NHS) Lothian, approval number 15‐HV‐016). The Edinburgh Brain Bank is a Medical Research Council funded facility with Research Ethics Committee (REC) approval (16/ES/0084).

Cryopreserved *post‐mortem* brain tissue from 10 AD cases (Braak VI) and 10 controls were used in immunohistochemistry (Table S2); this tissue was provided by the Newcastle Brain Tissue Resource which is funded in part by a grant from the UK Medical Research Council (G0400074), by NIHR Newcastle Biomedical Research Centre awarded to the Newcastle upon Tyne NHS Foundation Trust and Newcastle University, and as part of the Brains for Dementia Research Programme jointly funded by Alzheimer's Research UK and Alzheimer's Society.

### 2.4 Antibody generation

Mouse mAb to clusterin were generated by immunization of C57BL/6 mice (bred in‐house; supplementary methods) with native human clusterin using standard schedules.^42^, ^43^ Clusterin was immunoaffinity purified in‐house as described below. Immunized mice were screened for antibody responses by enzyme‐linked immunosorbent assay (ELISA), mice with the highest titer response were selected and re‐boosted before killing and harvesting of spleens. Plasma cells were harvested, fused with SP2 myeloma, and cultured in 96‐well plates. Positive hybridomas were selected by direct ELISA on immobilized clusterin (supplementary methods); clusterin‐positive mAb‐secreting clones were sub‐cloned by limiting dilution to monoclonality. Mouse mAb were iso‐typed using IsoStrips (Roche, #11493027001).

### 2.5 Immunoaffinity purification and functional characterization of clusterin

Anti‐clusterin mAb 2D5 (7 mg) was coupled to a HiTrap N‐hydroxysuccinimide (NHS) ‐activated high performance (HP) affinity column (Cytiva, #17071601). Human plasma was applied, the column washed, and bound protein eluted using 10 mM glycine (pH 2.5). Contaminating immunoglobulin G (IgG) was removed by incubation with protein A agarose (Thermo Fisher Scientific, #20333) and final polishing was by size exclusion chromatography (Superdex 200 Increase 10/300 GL; Cytiva, #28990944). Protein concentrations were determined by BCA assay.

The ability of clusterin to inhibit complement‐induced hemolysis was assessed in classical and reactive lysis assays, the former using sheep erythrocytes (TCS Biosciences, #SB069) sensitized with Siemens Amboceptor (Cruinn Diagnostics, #ORLC25; 1:4,000; ShEA) in HBS containing calcium and magnesium ions.^44^ ShEA (2% v/v) were added (50 µl/well) to a serial dilution of clusterin (50 µl/well; 100–0.2 µg/ml in duplicate in HBS) in a 96‐well round‐bottom plate; normal human serum (2.5% in HBS, 50 µl/well) was added and plates incubated at 37°C (30 minutes). After centrifugation (800 × *g*, 5 minutes, 4°C), absorbance at 405 nm was measured in supernatants and percent hemolysis calculated.

Complement terminal pathway inhibition by clusterin was tested using reactive lysis.^43^ Guinea pig erythrocytes (TCS Bioscience, #PB030AP) were diluted to 1% (v/v) in HBS and added (50 µl/well) to a serial dilution of clusterin (50 µl/well; 100–0.2 µg/ml in HBS) in a 96‐well round‐bottom plate. Terminal pathway components (all in‐house) were sequentially added (12.5 µl/well) and incubated at 37°C (10 minutes) prior to addition of the next component. Final concentrations in a 150 µl reaction mixture were: 45 ng/ml C5b6, 184 ng/ml C7, 168 ng/ml C8, 383 ng/ml C9. Following addition of C9, the plate was incubated at 37°C (30 minutes), plates were centrifuged and absorbance measured as above.

To test whether anti‐clusterin mAbs could prevent terminal pathway inhibition by clusterin, HBS containing 50 µg/ml clusterin and 100 µg/ml test mAb (50 µl/well) was added to GpE (50 µl/well) prior to addition of terminal pathway components as above (total volume 150 µl/well).

### 2.6 Characterization of mAbs and development of a sensitive and specific clusterin ELISA

To identify an optimal pair of mAbs for generating a quantitative sandwich ELISA, all eight selected mAbs were paired as capture or detection. Immunosorbent 96‐well plates (Thermo Fisher Scientific, #442404) were coated with capture mAbs (2 µg/ml, 50 µl/well) overnight at 4°C in carbonate buffer (35 mM NaHCO_3_, 15 mM Na_2_CO_3_, pH 9.6), blocked with PBS containing 0.05% (v/v) Tween‐20 (PBS‐T) and 2% (w/v) BSA for 30 minutes (37°C), washed with PBS‐T, then incubated with clusterin diluted from 1–0.0625 µg/ml in PBS‐T with 0.2% (w/v) BSA (dilution buffer; 50 µl/well) for 1 hour (37°C). Plates were washed and bound clusterin detected with each of the mAbs biotinylated using EZ‐Link Sulfo‐NHS‐LC‐Biotin (Thermo Fisher Scientific, #21327); 2 µg/ml in dilution buffer, 50 µl/well, for 1 hour at 37°C. Plates were washed, incubated with horseradish peroxidase (HRP) ‐conjugated streptavidin (R&D Systems, #DY998) diluted 1:200 in dilution buffer (50 µl/well) for 1 hour at 37°C, washed, developed with 50 µl/well O‐phenylenediamine dihydrochloride (SIGMAFAST™ OPD; SigmaAldrich, #P9187) and quenched with an equal volume of 5% (v/v) H_2_SO_4_. Absorbance was measured at 492 nm (Tecan, #30190077), response curves constructed and the mAb pair that showed the highest signal/noise ratio, mAb 2D5 as capture and biotinylated mAb 4C7 as detect, selected for further optimization.

The affinity of the selected mAbs was measured using surface plasmon resonance (SPR) on a Biacore T200 (Cytiva). Clusterin (92.6 RU) was immobilized on a Series S CM5 sensor chip (Cytiva, #29104988) using an amine coupling kit (Cytiva, #BR100050); R_max_ was kept low to account for mass transfer effects. The mAbs were diluted (100‐6.25 nM) in HBS‐P (10 mM HEPES, 150 mM NaCl, 0.05% Tween‐20, pH 7.4) and flowed (25°C, flow rate 30 µl/min) over the immobilized clusterin for 180 seconds, monitoring dissociation for 600 seconds, then regenerating with 10 mM glycine (pH 1.5) for 60 seconds. Data were collected, reference cell data subtracted to control for bulk refractive index (RI) changes and evaluated using Biacore Evaluation Software (Cytiva). Kinetics were calculated using the Langmuir 1:1 binding model; R_max_ and RI parameters were global and constant, respectively.

The optimized sandwich ELISA comprised mAb 2D5 as capture (5 µg/ml), clusterin standards (2 µg/ml–31.25 ng/ml), and biotinylated mAb 4C7 as detect (2 µg/ml). Clusterin was measured in plasma (diluted 1:500), sera (diluted 1:500), CSF (diluted 1:25), and astrocyte conditioned medium (undiluted, reported as ng/mg total protein). Upper and lower limits of detection were determined from an extended clusterin standard curve (10 µg/ml–0.3 ng/ml). Assay linearity was determined by measuring clusterin levels in plasma diluted 1:200‐1:3,200 (*n =* 5). Spike recovery was performed by measuring plasma with or without doping with 100 µg/ml clusterin and calculating percentage recovery (*n =* 5). Inter‐ and intra‐assay coefficients of variability (%CV) were calculated for plasma (*n =* 15) from healthy controls (median age: 30, range: 22–70; *n =* 9 male, *n =* 6 female) measured across three independent experiments.

### 2.7 Immunostaining of brain tissue and brain cells

Antibody selection and optimization of brain tissue immunostaining was performed using AD primary auditory cortex cryosections (10 µm) fixed in 4% (w/v) paraformaldehyde (PFA) and permeabilized in PBS with 0.1% (v/v) Triton X‐100 (TxPBS) containing 3% (w/v) BSA and 2% (w/v) normal goat serum (NGS; Vector Labs, #S‐1000). Sections were stained with anti‐clusterin mAbs (2D5 or 4C7, 5 µg/ml), and rabbit anti‐glial fibrillary acidic protein (GFAP) polyclonal antibody (pAb; 1:5,000; Dako, #GA524) or 6E10 rabbit anti‐Aβ mAb (1:500; Enzo Life Sciences, #ENZ‐ABS612‐0200) in TxPBS overnight (4°C). Isotype controls included mouse mAb IgG2a (BioLegend, #401501), mouse mAb IgG2b (BioLegend, #400301), and rabbit IgG (Invitrogen, #02‐6102). Sections were washed three times with TxPBS, then stained with 4ʹ,6‐diamidino‐2‐phenylindole (DAPI; 1:1,000; Merck), AlexaFluor 488‐conjugated goat anti‐rabbit IgG (1:1,000; Invitrogen, #A11008) and AlexaFluor 594‐conjugated goat anti‐mouse IgG (1:1,000; Invitrogen, #A32742) in TxPBS for 1 hour (room temperature [RT]). Sections were washed, endogenous autofluorescence quenched with 0.1 % (w/v) Sudan Black B (Alfa Aesar) in 70% (v/v) ethanol, mounted in FluorSave (Millipore, #345789), and imaged using the SP8 Lightning confocal microscope (Leica Biosystems). The optimal conditions were then used for quantitative clusterin immunostaining of hippocampal tissue. Cryosections (10 µm) were fixed, blocked, permeabilized, and co‐stained with 4C7 mouse anti‐clusterin mAb (3.13 µg/ml) and rabbit anti‐GFAP pAb (1:5,000) in immunohistochemistry (IHC) blocking buffer overnight at 4°C. Sections were washed and incubated with AlexaFluor 488‐conjugated goat anti‐rabbit IgG (1:1,000) and AlexaFluor 594‐conjugated goat anti‐mouse IgG (1:1,000) in IHC blocking buffer for 1 hour at RT. Sections were then washed and incubated with biotinylated 6E10 (1:1,000; Biolegend, #803007) in blocking buffer overnight at 4°C, then washed and incubated with AlexaFluor 647‐conjugated streptavidin (1:1,000; Invitrogen, #S21374) and DAPI (1:500) for 1 hour at RT. Autofluorescence quenching and imaging were performed as above. Clusterin‐positive astrocytes were quantified in hippocampal sections (control *n =* 10, AD *n =* 10; two sections/sample; four images per section) imaged as above (20× objective; 10 × 1 µm planes; laser power, gain and detection windows kept constant across images). The proportion and staining intensity of GFAP‐positive astrocytes co‐localizing with clusterin and the mean intensity of plaque proximal (< 20 µm) versus plaque distal (> 20 µm) clusterin‐positive astrocytes (in AD samples) was quantified in Imaris (version 10, Bitplane). In this latter analysis, plaque size was categorized according to the length of the Bounding Box OO (objected‐oriented) C‐axis as small (0–50 µm), medium (50–100 µm), or large (> 100 µm).

To test co‐localization of clusterin with synapses, cryosections were incubated with 2D5 or 4C7 mouse anti‐clusterin mAb (both at 3.13 µg/ml), chicken anti‐Homer1 pAb (1:1,000; Synaptic Systems, #160006), and guinea pig anti‐Bassoon mAb (1:1,000; Synaptic Systems, #141318) in blocking buffer overnight at 4°C. Sections were washed, incubated with AlexaFluor 488‐conjugated goat anti‐mouse IgG (1:1,000; Invitrogen, #A11001), AlexaFluor 594‐conjugated goat anti‐guinea pig IgG (1:1,000; Invitrogen, #A11076), and AlexaFluor 647‐conjugated anti‐chicken IgY (1:1,000; Invitrogen, #A21449) secondary antibodies in blocking buffer for 1 hour at RT. Autofluorescence quenching was performed as above, then sections were incubated with 1× Amylo‐Glo Amyloid Plaque Stain (Biosensis, #TR‐300‐AG) in 0.9% (w/v) saline for 10 minute at RT, washed, mounted, and imaged as above. For quantification of clusterin‐positive synaptic puncta, hippocampal sections (control *n =* 10, AD *n =* 10; two sections/sample; four images per section) were imaged as above, images cropped into 20 µm^2^ regions (five crops/image; including plaques in AD sections), imported into Imaris for batch‐processing and analysis. Total synapse number was obtained from Bassoon‐positive puncta (pre‐synaptic pan‐synapse marker), mature synapses were defined by puncta co‐staining for Homer1 (mature post‐synaptic marker) and Bassoon; clusterin‐positive total and mature synapses were defined by co‐staining with 4C7 anti‐clusterin and Bassoon, or Bassoon and Homer1, respectively.

Immunostaining of 1321N1 astrocytoma cells and iPSC‐derived astrocytes was performed in glass‐bottom black 96‐well plates, precoated with Geltrex for the latter. Cells (20,000/well) were washed, fixed in 4% PFA, blocked, permeabilized, and stained with rabbit anti‐GFAP (1:500) and mouse anti‐clusterin mAb (2D5 or 4C7; 5 µg/ml in PBS) overnight at 4°C. Isotype controls were included as above. Cells were washed then stained with DAPI, AlexaFluor 488‐conjugated goat anti‐mouse IgG (1:1,000), and AlexaFluor 594‐conjugated goat anti‐rabbit IgG (1:1,000; Invitrogen, #A32740) for 1 hour at RT. Cells were imaged as above.

### 2.8 Statistical analyses

All statistical analyzes were performed in Prism (GraphPad, version 11). All data were first tested for normality (Shapiro–Wilk test) and the appropriate test then applied. Two‐way analysis of variance (ANOVA) with Šidák's multiple comparisons test was used to compare reactive lysis inhibition by clusterin in the presence of anti‐clusterin mAbs relative to isotype controls. Paired *t*‐tests were used to compare clusterin levels in matched sera and plasma from the same donors, and densitometric signal between total brain homogenate (TBH) and synaptoneurosomes from the same brain samples. Clusterin/GFAP‐positive astrocytes in control and AD tissue were compared via Mann‐Whitney test; males and females were separately analyzed by unpaired *t‐*test and Welch's *t*‐test, respectively. Spatial analysis of clusterin/GFAP‐positive astrocytes relative to plaques in AD tissue was performed with the Wilcoxon matched‐pairs signed‐rank test; males and females were separately analyzed by Wilcoxon test and paired *t*‐test, respectively. Plaque‐associated clusterin in AD samples was stratified by plaque size and analyzed with Friedman's test with Dunn's multiple comparisons test. Unpaired *t‐*tests were used to compare mature and total clusterin‐positive synapses between control and AD tissue; males and females were separately analyzed via unpaired *t*‐tests. Data were then stratified by *APOE* ε4 carrier status and re‐analyzed via one‐way ANOVA with Tukey's multiple comparisons test. Results are indicated on graphs as * = *p* < 0.05, ** = *p* < 0.01. Figure legends contain statistical information for each experiment and graph, including statistical tests performed, definition of error bars, and definition of n.

## 3 RESULTS

### 3.1 Clusterin mAb generation and characterization

Hybridoma clones were selected for further characterization based upon the capacity of clone supernatants to bind clusterin in direct ELISA (Figure S1); the mAbs were all either IgG2a (4C7) or IgG2b (1A4, 1D5, 2D5, 3C9, 5C11, 6C12, 6D1). Non‐competing mAb pairs suitable for generating a quantitative clusterin ELISA were identified by sandwich ELISA, with 2D5 capture and 4C7 detection performing best (Figure 1A); 4C7 was non‐competitive with all other seven mAbs, suggesting a unique epitope (Figure S2). Both 2D5 and 4C7 detected denatured clusterin in serum by Western blot (Figure 1B); mAbs 6C12 and 6D1 also performed well in Westerns (Figure S3). SPR analysis on immobilized clusterin confirmed strong binding of mAbs 2D5 (KD = 11.28 nM) and 4C7 (KD = 1.84 nM) (Figure 1C–E).

**FIGURE 1 alz71885-fig-0001:**
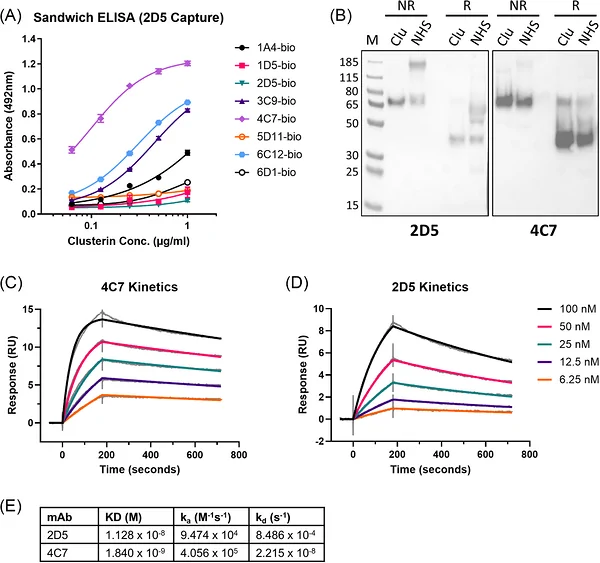
Characterization of novel anti‐clusterin mAbs. (A) Representative sandwich ELISA testing compatibility of mAb 2D5 as capture with all eight clusterin mAbs as detection. Purified clusterin was used as standard (1–0.0625 µg/ml), 2D5 and biotinylated detection mAbs were used at 2 µg/ml. Error bars represent standard error of duplicates; data for other capture mAbs can be found in Figure S2. (B) Western blot showing detection with mAbs 2D5 and 4C7 (2 µg/ml) of purified clusterin (80 ng/lane) and clusterin in serum (NHS; 1 µl/lane) run under NR (single band at 70 kDa) and R (double band at 40 kDa) conditions. Secondary only and other mAb blots are shown in Figure S3. (C–D) Representative SPR sensorgrams showing mAbs 2D5 and 4C7 (100–6.25 nM) binding clusterin immobilized on the surface. Gray and colored lines represent raw and fitted data, respectively. (E) Binding kinetics were calculated from three independent experiments; KD, k_a_, and k_d_ are the equilibrium dissociation constant, association rate constant, and dissociation rate constant, respectively. mAb, monoclonal antibody; ELISA, enzyme‐linked immunosorbent assay; NHS, N‐hydroxysuccinimide; NR, non‐reducing; R, reducing; SPR, surface plasmon resonance.

Clusterin purified in‐house from healthy donor plasma on a 2D5 immunoaffinity column was 84% pure by densitometry with a single ∼20 kDa contaminant in both non‐reduced and reduced conditions; neither size‐exclusion chromatography nor incubation with protein A resin removed this contaminant, implying that it was tightly but non‐covalently associated with clusterin (Figure 2A). Immunoaffinity‐purified clusterin efficiently bound the different mAbs in direct ELISA (Figure 2B) but did not inhibit complement‐induced lysis in a serum‐based classical pathway hemolytic assay (Figure 2C), likely due to the abundance of endogenous clusterin in serum and clusterin interacting with other serum proteins; however, efficient inhibition of membrane attack complex (MAC) assembly was observed in serum‐free reactive lysis assays (Figure 2D). None of the anti‐clusterin mAbs blocked MAC inhibition by clusterin in reactive lysis assays (Figure 2e).

**FIGURE 2 alz71885-fig-0002:**
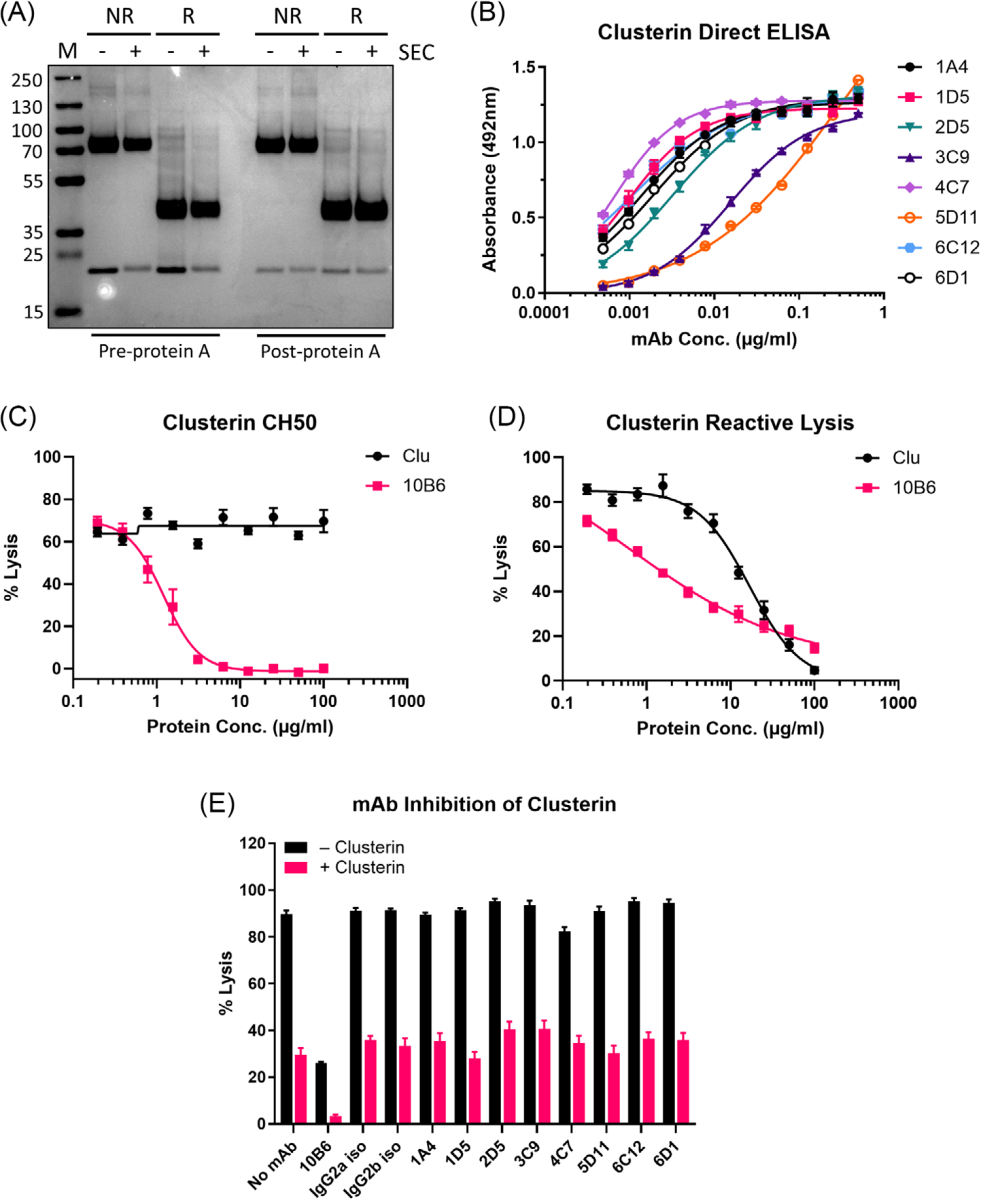
Characterization of mAb binding and function of immunoaffinity‐purified clusterin. (A) SDS‐PAGE analysis of 2D5‐immunoaffinity purified clusterin (5 µg/lane) under NR or R conditions and with (+) or without (‐) an SEC final step; bands were quantified by densitometry. (B) Representative direct ELISA displaying binding of anti‐clusterin mAbs (0.5 µg/ml–0.5 ng/ml) to 2D5‐immunoaffinity purified clusterin (0.5 µg/ml) immobilized on plastic; error bars represent standard error of duplicates. (C) Clusterin does not inhibit hemolysis in classical pathway assay compared to blocking anti‐C5 mAb 10B6 as positive control. (D) Clusterin shows efficient inhibition of hemolysis in a reactive lysis assay, with 10B6 as positive control. (E) None of the anti‐clusterin mAbs, added at two‐fold stoichiometric excess compared to clusterin, blocked the inhibitory activity of clusterin in reactive lysis assays. Isotype controls were used for comparison and data were analyzed using a two‐way ANOVA with Šidák's test (*p >* 0.05). In each plate, error bars represent standard error of three independent experiments performed in duplicate. ANOVA, analysis of variance; ELISA, enzyme‐linked immunosorbent assay; mAb, monoclonal antibody; NR, non‐reducing; R, reducing; SDS‐PAGE, sodium dodecyl sulfate‐polyacrylamide gel electrophoresis; SEC, size exclusion chromatography.

### 3.2 Development of clusterin sandwich ELISA

A quantitative sandwich ELISA was developed to measure clusterin in human biofluids comprising mAb 2D5 as capture (5 µg/ml) and biotinylated mAb 4C7 as detection (2 µg/ml). The assay lower limit of detection was 5 ng/ml, upper limit of detection was 5 µg/ml (Figure 3A). Mean spike recovery, assessed by doping five plasma samples with 100 µg/ml clusterin and measuring clusterin levels relative to un‐doped samples, was 83.6% (Figure 3B). Assay linearity was confirmed by interpolating serial 1:2 serial dilutions (1:200–1:3,200) of five plasma samples on the standard curve with the resultant data shown in the table; CVs across the dilutions were < 10% for each plasma sample (Figure 3C). Normal range in plasma and assay reproducibility was assessed by measuring clusterin in 15 plasma samples in three separate experiments; across the three assays mean plasma clusterin was 147.6 µg/ml (range: 113.3–185.3 µg/ml), intra‐assay CV was 3.93%, and inter‐assay CV was 8.5% (Figure 3D). To compare levels in plasma and serum and test the capacity of the assay to quantify clusterin in CSF, 11 matched plasma and serum samples and 10 unmatched CSF samples were measured in the assay. Comparison of matched plasma and serum revealed a small but not significant reduction in clusterin levels measured in serum (mean: 149.0 µg/ml, range 106.8–199.0 µg/ml) compared to plasma (mean: 165.3 µg/ml, range: 139.5–216.4 µg/ml); levels in CSF were approximately 5% of those in plasma (mean: 6.76 µg/ml, range 2.28–12.41 µg/ml) (Figure 3E). The assay did not detect clusterin in mouse or rat sera (Figure S4A); in Western blot analysis of rodent sera 2D5 recognized both mouse and rat clusterin, while 4C7 displayed poor cross‐reactivity (Figure S4B).

**FIGURE 3 alz71885-fig-0003:**
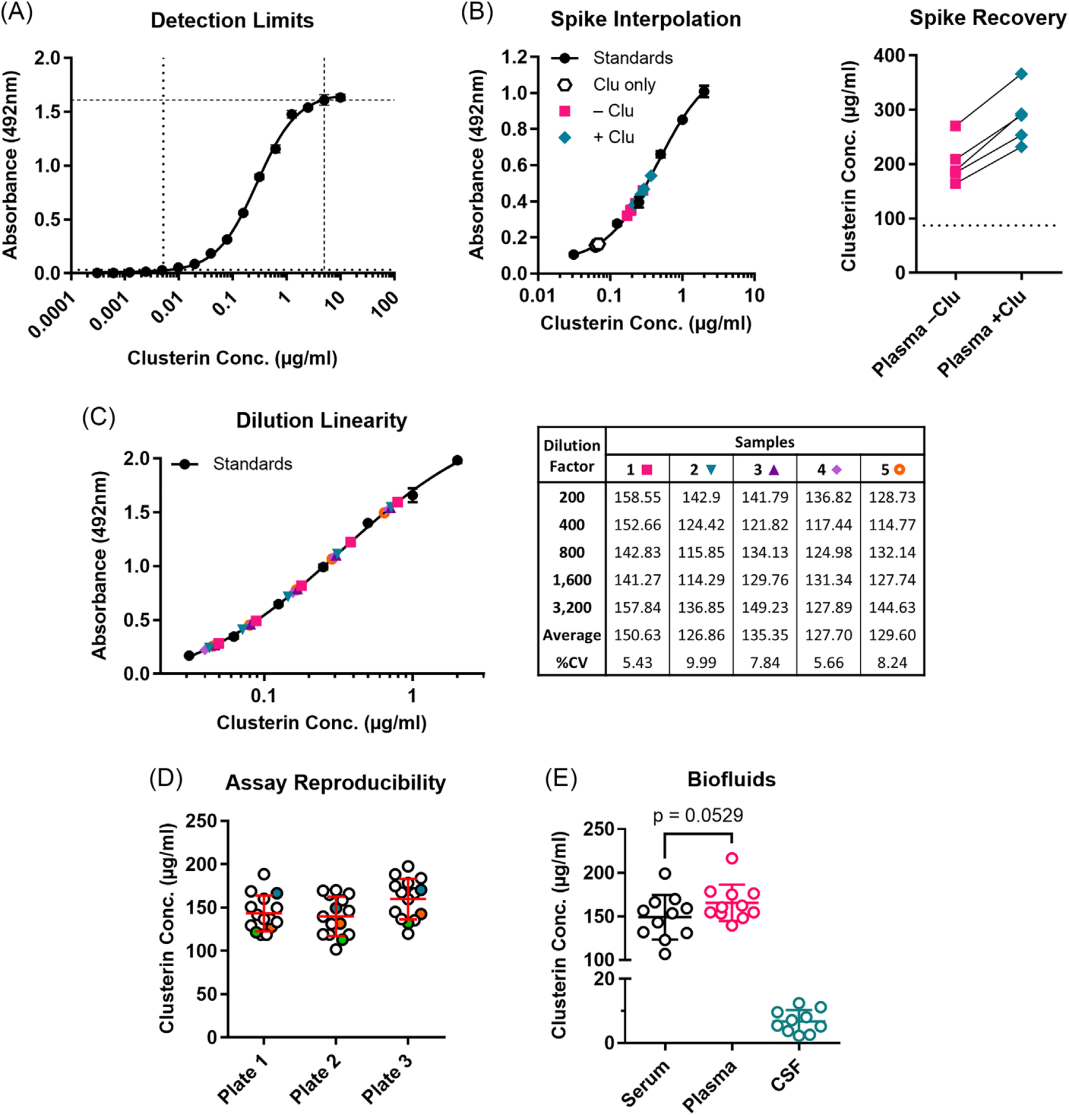
Validation of in‐house clusterin sandwich ELISA. (A) ELISA standard curve (10 µg/ml–0.3 ng/ml) displaying upper (5 µg/ml) and lower (5 ng/ml) detection limits and a three‐log assay working range. (B) Spike recovery experiment displaying clusterin ELISA measurement in five plasma samples with and without supplementation with 100 µg/ml clusterin, mean recovery of the supplemented dose was 83.6%. The dotted line represents assay buffer supplemented with 100 µg/ml clusterin only, recovery was 87.0%. (C) Dilution linearity was tested in five plasma samples serially diluted (1:200–1:3,200) prior to measurement in the ELISA, sample concentrations are reported in µg/ml. (D) Intra/inter‐assay CVs were calculated by measuring the same set of 15 plasma samples in three separate experiments; intra‐assay CV was 3.93%, inter‐assay CV was 8.5%. Values of three representative samples across the three assays are colored to further illustrate reproducibility. (E) Clusterin was measured in unmatched CSF (*n =* 10) and matched serum and plasma (*n =* 11), no significant difference was observed between matched serum and plasma (paired *t*‐test, *p =* 0.0529). Error bars represent standard error of duplicates in (A–C) and sample set mean and standard deviation in (D,E). CSF, cerebrospinal fluid; CV, coefficients of variation; ELISA, enzyme‐linked immunosorbent assay.

### 3.3 Detection of clusterin in human brain tissue and iPSC‐derived astrocytes

To assess whether the novel mAbs detected endogenous clusterin in brain tissues, we performed immunohistochemistry and Western blotting on human AD brain. Both mAb 2D5 and mAb 4C7 strongly co‐localized with Aβ plaques; 4C7 but not 2D5 also labeled GFAP‐positive astrocytes in a cytosolic pattern, suggesting that while 2D5 and 4C7 both detect plaque labeling by secreted clusterin, only 4C7 detected intracellular clusterin (Figure 4A). Western blotting of healthy control brain tissue with mAb 4C7 detected clusterin in TBH and isolated synaptoneurosomes, slightly more in TBH compared to matched synaptoneurosomes (Figure 4B,C).

**FIGURE 4 alz71885-fig-0004:**
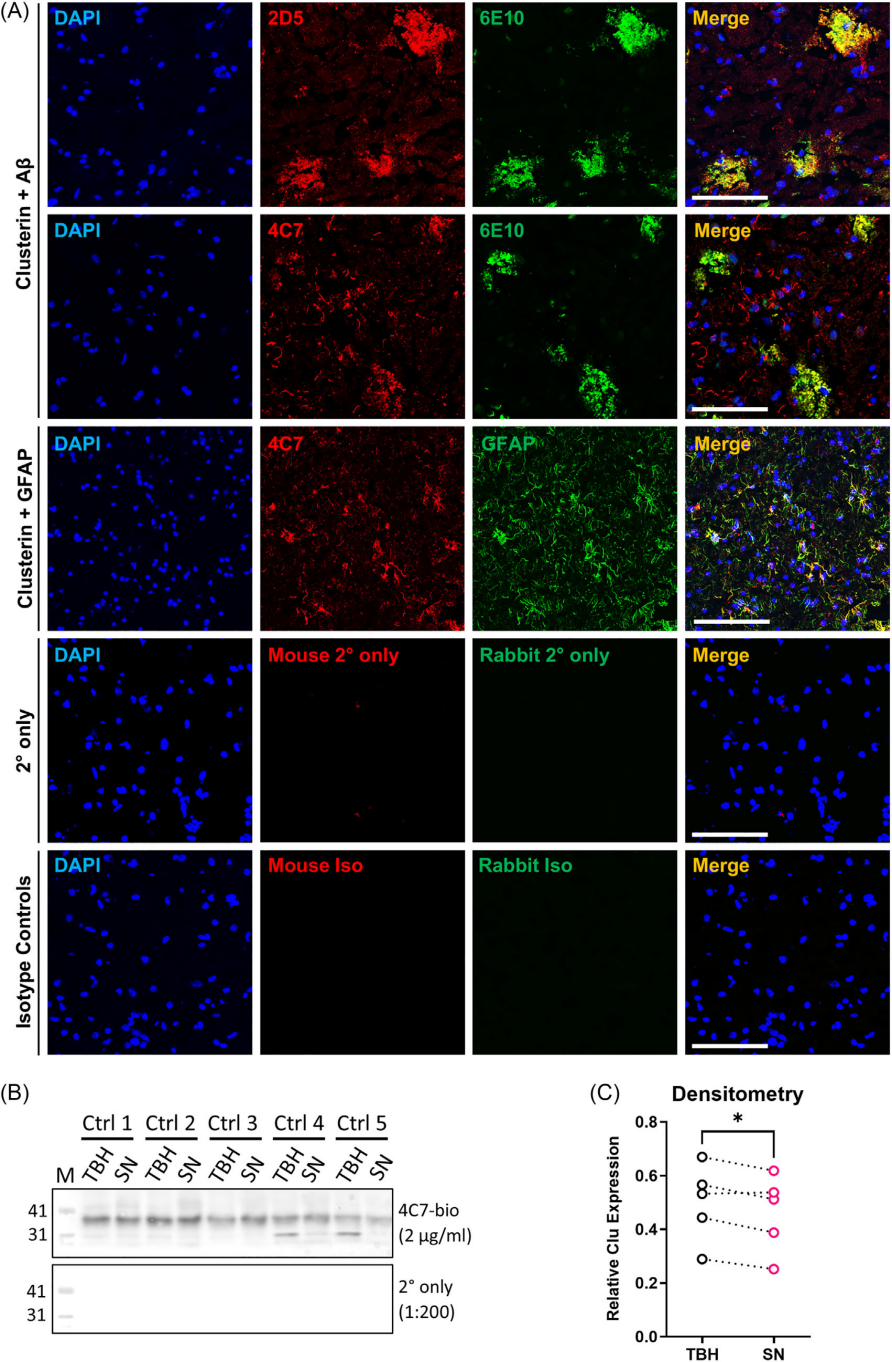
Detection of clusterin in human brain tissue using novel mAbs that differentiate clusterin isoforms. (A) Representative immunostaining of human AD primary auditory cortex (SD037/18) with mAb 2D5 or 4C7 (column 2, red) co‐stained with 6E10 anti‐Aβ RabmAb (column 3, green) is shown in the top two rows; co‐staining with mAb 4C7 (red) and rabbit anti‐GFAP pAb (column 3, green) is shown in the middle row. Secondary (2°) only and isotype controls are in the bottom two rows and blue DAPI staining in column 1. Merged images are in column 4 and include 100 µm scale bars. (B) Western blot detection of clusterin in healthy control TBH and SN) samples under reducing conditions (10 µg/lane), including streptavidin‐HRP secondary (2°) only control blot. Uncropped Westerns are shown in Figure S5. (C) Densitometry of clusterin signal in Western blots normalized to total protein in paired TBH and SN samples (*n =* 5; Table S1); dashed lines show paired data from the same tissue sample; a paired *t*‐test was used to calculate significance (*p =* 0.029). AD, Alzheimer's disease; DAPI, 4′,6‐diamidino‐2‐phenylindole; GFAP, glial fibrillary acidic protein; pAb, polyclonal antibody; SN, synaptoneurosome; TBH, total brain homogenate.

To confirm the observed astrocyte expression of clusterin in brain, we tested mAb 2D5 and 4C7 staining of the 1321N1 astrocytoma cell line and iPSC‐derived astrocytes; strong intracellular clusterin staining with mAb 4C7 was detected after permeabilization in both cell types; negligible staining was observed with 2D5 on permeabilized cells, replicating the brain staining findings and strengthening the assertion that 4C7 but not 2D5 detected intracellular clusterin (Figure 5A,B). Expression of transcripts specific for intracellular and secreted *CLU* was confirmed in five lines of iPSC‐derived astrocytes (Figure 5C–E). Secreted clusterin protein was detected by ELISA in conditioned media from each of the iPSC‐derived astrocyte lines, albeit with substantial variability between the lines (Figure 5F). Notably, three of the five lines showed very low levels of secreted clusterin in this assay.

**FIGURE 5 alz71885-fig-0005:**
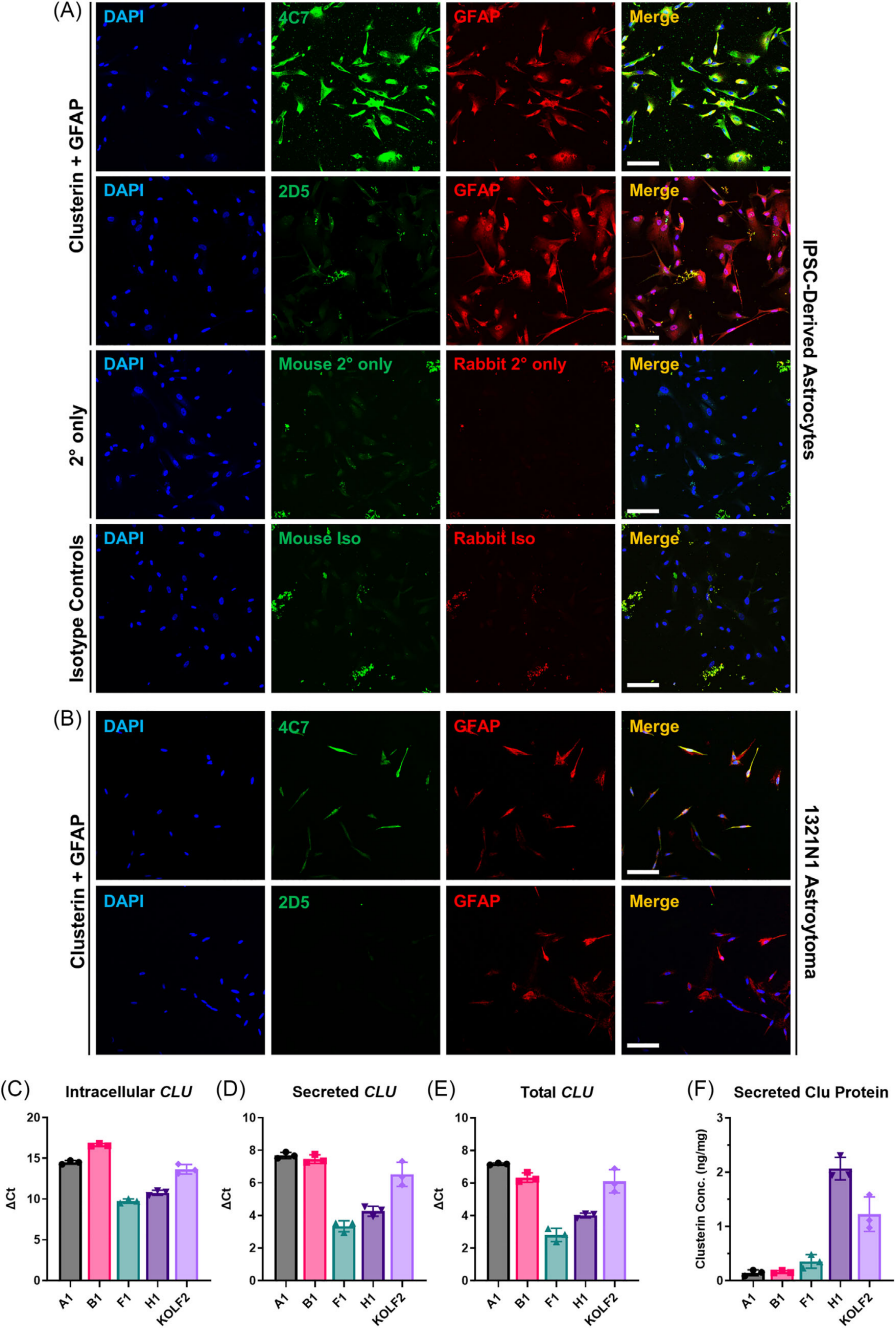
Detection of clusterin in astrocytoma cells and iPSC‐derived astrocytes using novel mAbs that differentiate clusterin isoforms. (A) Representative immunostaining of permeabilized human iPSC‐derived astrocytes with anti‐clusterin mAbs 2D5 and 4C7 (column 2, green), co‐stained with rabbit anti‐GFAP pAb (column 3, red). Secondary only and isotype controls are in the bottom two rows and blue DAPI staining in column 1. Merged images are in column 4 and include 100 µm scale bars. (B) Representative immunostaining of permeabilized 1321N1 astrocytoma cells with mAb 2D5 or 4C7 (column 2, green) co‐stained with rabbit anti‐GFAP pAb (column 3, red). Blue DAPI staining is in column 1; merged images are in column 4 and include 100 µm scale bars. RT‐qPCR quantification of *CLU* mRNA encoding (C) intracellular, (D) secreted, and (E) all isoforms of clusterin in five iPSC‐derived astrocyte lines. Data are normalized to *GAPDH* and *ACTB* expression for each cell line; error bars represent standard error of triplicates. RT‐qPCR methods can be found in supplementary methods and information on primers is shown in Table S3. (F) ELISA measurement of clusterin concentration in conditioned medium prepared from five iPSC‐derived astrocyte lines; error bars represent standard error of triplicates. DAPI, 4′,6‐diamidino‐2‐phenylindole; ELISA, enzyme‐linked immunosorbent assay; GFAP, glial fibrillary acidic protein; iPSC, induced pluripotent stem cell; mAb, monoclonal antibody; pAb, polyclonal antibody; RT‐qPCR, reverse transcriptase‐quantitative polymerase chain reaction.

### 3.4 Clusterin is enriched in plaque‐associated astrocytes in the AD hippocampus

To investigate the distribution of clusterin in the hippocampus, immunofluorescence staining of tissue from AD cases (Braak stage VI) and age‐matched controls was performed, staining for clusterin (4C7 or 2D5), astrocytes (GFAP), amyloid plaques (6E10), and nuclei (DAPI). Both of the anti‐clusterin mAbs strongly and ubiquitously stained plaques, while mAb 4C7 but not 2D5 also stained GFAP‐positive astrocytes in a cytoplasmic pattern, demonstrating that mAb 4C7 stained both secreted and intracellular clusterin (Figure 6A,B). Quantitative analysis of 4C7/GFAP co‐staining revealed no significant difference in clusterin staining of GFAP‐positive astrocytes between controls and AD cases (Figure 6C); however, spatial analysis within AD samples demonstrated a significant increase in clusterin signal in astrocytes located proximal (< 20 µm) to amyloid plaques compared to those distal (> 20 µm) to plaques (Figure 6D). Further stratification by plaque size showed that clusterin‐positive astrocyte signal was significantly increased in association with larger plaques (> 100 µm) compared to smaller plaques (< 50 µm) (Figure 6E).

**FIGURE 6 alz71885-fig-0006:**
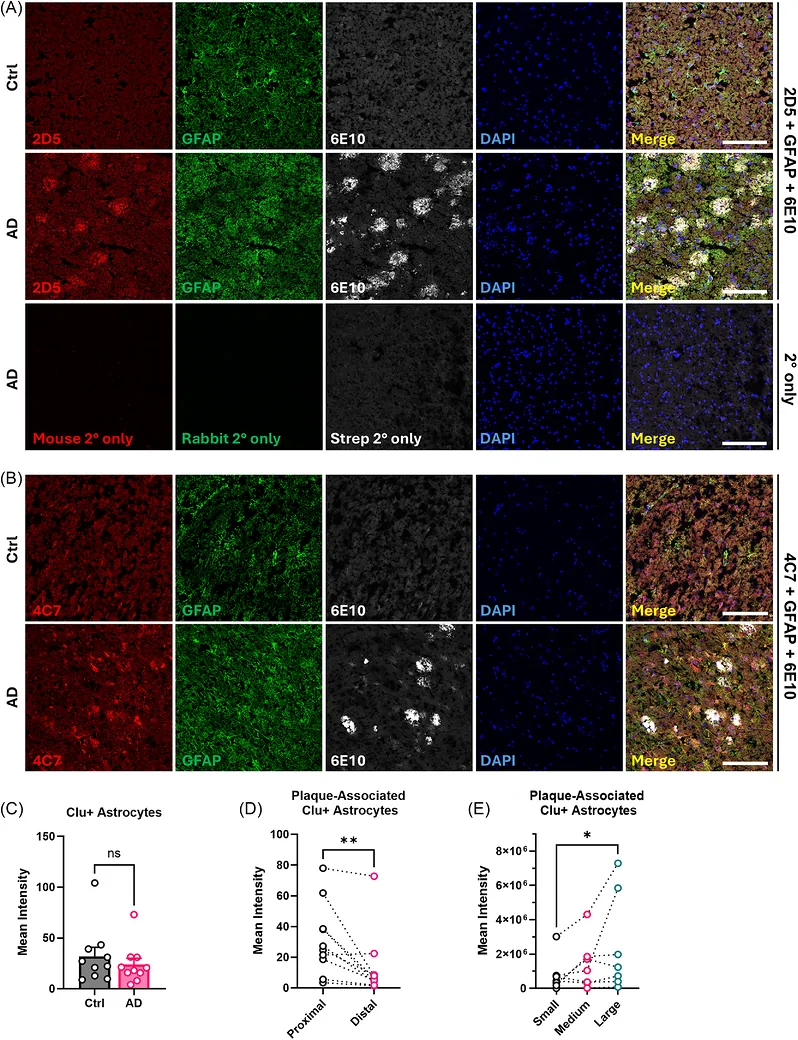
Mapping clusterin expression in astrocytes in AD and control hippocampus. (A) Representative confocal images of human hippocampal cryosections from Ctrl and AD tissue stained for clusterin (2D5, red), astrocytes (GFAP, green), amyloid plaques (6E10, gray), and nuclei (DAPI, blue). Merged images show the localization of clusterin to plaques, scale bars are 100 µm; the bottom row is secondary (2°) only control staining of AD tissue. (B) Representative confocal images of human hippocampal cryosections from controls (Ctrl) and AD cases (*n =* 10 for each; two sections per case, four images per section; Table S2), stained for clusterin (4C7, red), astrocytes (GFAP, green), amyloid plaques (6E10, gray), and nuclei (DAPI, blue). Merged images show the localization of clusterin to astrocytes and plaques; scale bars are 100 µm. (C) Clusterin (4C7) co‐localized with GFAP‐positive astrocytes with no significant difference in staining intensity between AD and control cases (*n =* 10 for cases and controls; Mann–Whitney test, *p >* 0.05); error bars represent standard error. Sex‐disaggregated data can be found in Figure S6a. (D) Quantification of GFAP/clusterin‐positive astrocytes proximal (< 20 µm) and distal (> 20 µm) to plaques in individual AD samples, linked by dotted lines, revealed increased signal in plaque‐proximal regions (*n =* 10 cases and controls; Wilcoxon test, *p =* 0.0039). Sex‐disaggregated data can be found in Figure S6b. (**e**) Quantification of plaque‐associated GFAP/clusterin‐positive astrocytes in individual AD samples (linked by dotted lines), stratified by plaque size (small: < 50 µm, medium: 50–100 µm, large: > 100 µm), showed significantly increased signal associated with large compared to small plaques (Friedman test with Dunn's test, *p =* 0.0485). Three of the 10 AD samples did not have large plaques so could not be included in this analysis; sex‐disaggregated data analysis could not be performed due to low sample numbers. AD, Alzheimer's disease; Ctrl, control; DAPI, 4′,6‐diamidino‐2‐phenylindole GFAP, glial fibrillary acidic protein.

### 3.5 Clusterin localizes to the surface of synapses

To assess whether clusterin is expressed at synapses, hippocampal sections were co‐stained with anti‐clusterin mAbs (4C7 or 2D5) and pre‐ and post‐synaptic markers (Bassoon and Homer1, respectively). High‐resolution imaging demonstrated co‐localization of clusterin (stained with either 4C7 or 2D5) with synaptic puncta in both control and AD tissue (Figure 7A,B), confirming its presence at synapses and verifying the Western blot data (Figure 4B). The synaptic staining patterns obtained with mAb 4C7 and 2D5 were identical, indicating that the mAbs are detecting clusterin localized on the surface of synapses. Quantification of the proportion of clusterin‐positive total (Bassoon‐positive puncta) and mature (Bassoon/Homer1 co‐localized puncta) synapses revealed no significant difference between controls and AD cases (Figure 7C,D); stratification by *APOE* ε4 status did not reveal any significant differences in clusterin staining at synapses (Figure 7E,F).

**FIGURE 7 alz71885-fig-0007:**
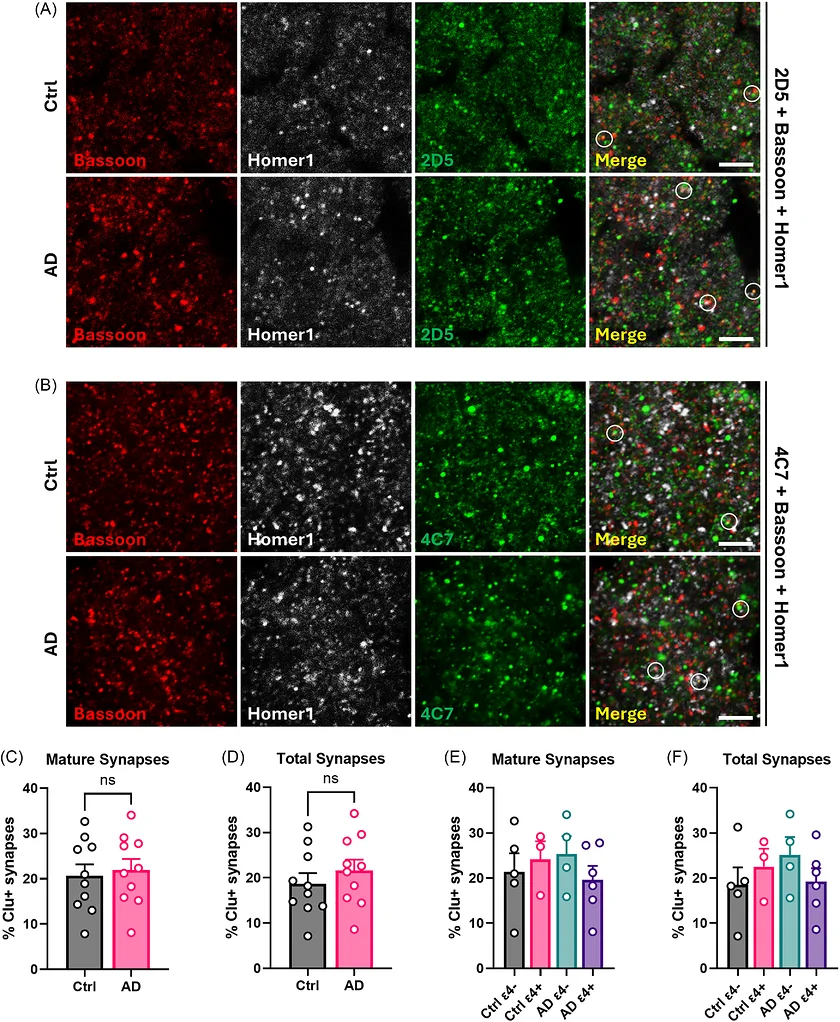
Mapping clusterin expression in synapses in AD and control hippocampus. (A) Representative confocal images of human hippocampal cryosections from Ctrl and AD tissue showing co‐localization of clusterin (2D5, green) with the pre‐synaptic marker Bassoon (red) and mature post‐synaptic marker Homer1 (gray). (B) Representative confocal images of human hippocampal cryosections from controls (Ctrl) and AD cases (*n =* 10 for each; two sections per case, four images per section, Table S2), stained for clusterin (4C7, red), astrocytes (GFAP, green), amyloid plaques (6E10, gray), and nuclei (DAPI, blue). Merged images in (A,B) confirm co‐localization of clusterin with synaptic markers, scale bars are 4 µm; white circles indicate examples of co‐localization. (C) Quantification of the percentage of mature synapses (puncta positive for both Bassoon and Homer1) positive for clusterin (mAb 4C7) showed no significant difference between Ctrl and AD samples (*n =* 10 cases and controls; unpaired *t*‐test, *p >* 0.05). Sex‐disaggregated data can be found in Figure S6C. (D) Quantification of the percentage of total synapses (Bassoon‐positive puncta) co‐localized with clusterin (4C7) showed no significant difference between Ctrl and AD groups (*n =* 10 per group; unpaired *t*‐test, *p >* 0.05). Sex‐disaggregated data can be found in Figure S6D. Quantification of the percentage of clusterin‐positive mature (**e**) or total (F) synapses showed no significant difference between Ctrl and AD samples regardless of *APOE* ε4 status (one *APOE* ε4 allele versus none; one‐way ANOVA with Šidák's test, *p >* 0.05). total clusterin‐positive synapses were defined as co‐localization of 4C7 staining with puncta positive for Bassoon and mature clusterin synapses as co‐localization with puncta positive for both Bassoon and Homer1. Data represent *n =* 5 controls with no *APOE* ε4 alleles (Ctrl ε4‐), *n =* 3 controls with at least one *APOE* ε4 allele (Ctrl ε4+), *n =* 4 AD cases with no *APOE* ε4 alleles (AD ε4‐), and *n =* 6 AD cases with at least one *APOE* ε4 allele (AD ε4+); two controls were excluded because *APOE* genotype was not recorded (Table S2). Error bars in all plates represent standard error. AD, Alzheimer's disease; ANOVA, analysis of variance; APO, apolipoprotein E; Ctrl, control; DAPI, 4′,6‐diamidino‐2‐phenylindole; GFAP, glial fibrillary acidic protein; mAb, monoclonal antibody.

## 4 DISCUSSION

Clusterin is ubiquitously expressed and has diverse roles, implicated in numerous biological pathways and playing roles in homeostasis and pathology. It has gained particular attention in AD where early GWAS identified risk variants in the *CLU* gene,^3^, ^4^ a finding replicated in several studies and supported by the implication of clusterin as a biomarker in AD.^31^, ^32^, ^35^ Variability between published biomarker studies likely reflects the lack of well‐characterized reagents and robust, sensitive, and specific assays, essential to ensure measurements are accurate and reproducible across different cohorts. To fill this gap, we generated a panel of clusterin‐specific mAbs and explored their specificity and utility for different applications. Two non‐competing mAbs with high affinity and specificity for human clusterin (2D5 and 4C7) were selected as the optimal combination for a sandwich ELISA to accurately quantify clusterin in human biological fluids and tissue extracts. While mAb 4C7 detected both secreted and intracellular clusterin isoforms, mAb 2D5 did not detect the cytoplasmic isoform, providing for the first time tools to differentiate these in cells and tissues.

Clusterin levels in human plasma, serum, and CSF were measured in samples from normal donors to establish assay performance and normal ranges in these different fluids. Both mAbs also detected clusterin by Western blotting in these fluids and in tissue extracts. The mAb 2D5, immobilized on Sepharose, isolated functional clusterin from human plasma in a single step, albeit with an unidentified 20 kDa contaminant that resisted attempts to remove by size exclusion chromatography, suggesting a strong association. Clusterin is a chaperone in plasma; hence, association and co‐purification with other plasma proteins is well‐described, with albumin, caeruloplasmin, fibrinogen and complement component C7 among the proteins identified in previous studies.^45^, ^46^ None of these are compatible with the observed contaminant. None of the mAbs blocked clusterin inhibition of MAC assembly, suggesting that their epitopes are not located on the surface of the helical domains that interact with MAC intermediate complexes.^47^, ^48^ The mAbs may inhibit clusterin interactions with other proteins, but we do not possess the functional assays required to investigate these mechanisms.

Although measurement of clusterin in CSF and/or plasma has been proposed as a biomarker of AD, results to date have been inconsistent.^49^, ^50^, ^51^ While many studies reported increased clusterin levels in AD plasma or CSF compared to healthy controls, others found no significant differences or even decreased levels; indeed, a meta‐analysis published in 2019 concluded that neither plasma nor CSF levels of clusterin were reliable biomarkers for AD.^52^ These inconsistencies likely reflect assay variability; the development and validation of a sensitive and specific clusterin assay should aid clarification of the utility of clusterin as an AD biomarker. Indeed, an early version of the assay, prior to optimization but using the same mAbs, was used to measure clusterin in a cohort comprising 1,500 AD patient and 500 control plasma samples and found significantly higher levels in AD patients, particularly in those with early onset (before 65 years) AD.^31^ Mean plasma clusterin in the control cohort was 195.8 µg/ml, higher than what we observed in healthy control plasma with the optimized assay in the present study. The early version of the assay was also used to measure clusterin in long COVID, myasthenia gravis, and Down syndrome cohorts;^53^, ^54^, ^55^ here, we describe the full validation of the anti‐clusterin mAbs and their use in the exploring roles of clusterin in AD.

The mAbs 2D5 and 4C7 both bound clusterin in biofluids and specifically stained clusterin marking amyloid plaques in AD brain tissue; however, staining of glia, specifically astrocytes, was seen only with 4C7. Notably, epitope binning of the eight anti‐clusterin mAbs demonstrated that 4C7 bound a unique epitope in clusterin. The observed astrocyte staining pattern suggested that mAb 4C7 bound intracellular clusterin in addition to secreted clusterin in situ in tissues, supported by demonstrating 4C7 cytoplasmic staining in an astrocyte line and iPSC‐derived astrocytes, observed only after cell permeabilization. Using isoform‐specific primers, we showed that all tested iPSC‐derived astrocytes expressed message for both secreted and intracellular clusterin; secreted clusterin was detected using the ELISA in medium from these cells. Co‐localization of clusterin with Aβ plaques in AD brain was described over thirty years ago,^16^, ^56^, ^57^, ^58^ but glial staining was not reported, suggesting that the reagents used in these studies detected only secreted clusterin. The combined use of mAbs 2D5 (binds only secreted clusterin) and 4C7 (also binds intracellular clusterin) provides a powerful tool for assessing clusterin localization in cells, tissues and fluids.

A comparison of staining patterns in a larger set of control and AD hippocampal tissue samples confirmed that mAb 4C7 robustly labeled GFAP‐positive astrocytes while 2D5 showed no staining. Both mAbs strongly and ubiquitously labeled plaques in AD tissue, confirming previous reports. While there was no difference in overall numbers of GFAP/clusterin‐positive astrocytes between control and AD tissue, clusterin‐expressing astrocytes were significantly more abundant in the vicinity of plaques in AD cases, particularly around larger plaques, suggesting a localized role in plaque‐related pathology. The mAb 4C7 also stained Bassoon‐positive synapses; mAb 2D5 stained synapses in an identical manner, demonstrating that the observed staining represented secreted clusterin localized to the surface of synapses. The presence of clusterin at synapses was supported by Western blotting of synaptic fractions; however, precisely what clusterin binds to at the synapse remains undefined. Approximately 20% of synapses in controls and AD cases were clusterin‐positive in hippocampus; as a first attempt to identify whether specific types of synapses preferentially bound clusterin, we utilized Homer1 staining of mature glutamatergic synapses. The proportion of clusterin‐positive synapses was similar for total and mature synapses and between control and AD hippocampus, indicating that clusterin does not selectively bind mature synapses; further exploration of synapse selectivity of clusterin staining is justified. Others reported clusterin localization to synapses in AD primary auditory cortex; in this study, the proportion of clusterin‐positive synapses was increased in AD cases compared to controls, but only in *APOE* ε4 carriers.^59^ This finding provoked us to stratify our AD cases and controls by *APOE* ε4 carrier status; we saw no significant differences, although this may be a consequence of our comparatively small cohort.

In summary, we have developed novel mAbs against human clusterin that enable accurate quantification of clusterin in human biofluids and tissues, and visualization of clusterin in brain tissue and cell lines. We demonstrated that the selected mAbs when used together enabled differentiation of secreted clusterin, including clusterin fixed in plaques and at synapses, from intracellular clusterin detected in astrocytes both in tissues and in culture. These mAbs have been licensed to Hycult Biotech (2D5, #HM2435; 4C7, #HM2436). Future work will leverage these tools to explore the relative contributions of secreted and intracellular clusterin to brain health and AD pathology.

## AUTHOR CONTRIBUTIONS

Aurora Veteleanu and Wioleta M. Zelek designed and led the antibody generation work. Aurora Veteleanu and Robert A.J. Byrne led the antibody characterization studies; Robert A. J. Byrne prepared the manuscript. ELISA characterization was performed by Olivia G. Bacon; Westerns by Hannah L. Cramp, Matthew Bright; SPR by Kok Yung Lee; immunocytochemistry by Hannah L. Cramp, Gareth D. Fenn, Mariana Flores Castelán. Clusterin purification and functional characterization was performed by Hannah L. Cramp, Rebekah S. Cooke. The clusterin sandwich ELISA was designed by Aurora Veteleanu and validated by Kirsten L. Baillie. Nikoleta Daskoulidou designed and led the human brain tissue and stem cell work. Nikoleta Daskoulidou, B. Paul Morgan, and Wioleta M. Zelek supervised the project and finalized the manuscript for submission. B. Paul Morgan, Wioleta M. Zelek and Nikoleta Daskoulidou: funding acquisition. All authors discussed the content and approved the final version of the manuscript.

## CONFLICT OF INTEREST STATEMENT

B. Paul Morgan serves as a Director, and Wioleta M. Zelek as a Scientific Advisor to Acionna Therapeutics. All other authors have nothing to disclose. Author disclosures are available in the Supporting Information.

## CONSENT STATEMENT

Human subjects were involved in this research; informed consent was obtained.

## Supporting information




Supporting Information

Supporting Information

## Data Availability

Information on the data underpinning the results presented here, including how to access them, can be found in the Cardiff University data catalogue at [10.17035/cardiff.32790288].
