# Supplementary material for: Clusterin is enriched in plaque‐associated astrocytes and localizes to synapses in Alzheimer's disease
Source: Alzheimers Dement. 2026 Oct 6;22(10):e71885. doi: 10.1002/alz.71885 (PMC13640977; doi:10.1002/alz.71885)
Supplement: Supplementary file 2 [file ALZ-22-e71885-s001.docx]

## Supplementary Figures

**Supplementary Figure 1: Generation of anti-clusterin mAbs.** (**a**) Coomassie staining of in-house clusterin used for immunisation under reducing (R) and non-reducing (NR) conditions (2.5 µg/lane; 12.5% gel). (**b**) Direct ELISA screening for anti-clusterin mAbs in immune sera from immunised mice. (**c**) Representative raw ELISA data displaying screening of hybridoma conditioned media against clusterin. Hybridoma clones that produced clusterin antibodies were recloned (black boxes). (**d**) Coomassie staining of purified clusterin mAbs (2.5 µg/lane) showing a 150 kDa band corresponding to IgG in non-reduced samples and 55 kDa (IgG heavy chain) and 25 kDa (IgG light chain) bands in reduced samples.

**Supplementary Figure 2: Testing clusterin mAbs for sandwich ELISA compatibility.** All eight clusterin mAbs were paired in sandwich ELISAs on purified clusterin (1–0.0625 µg/ml). Capture mAbs and biotinylated detection mAbs were both used at 2 µg/ml. Data for mAb 2D5 capture is shown in Figure 1a. Error bars represent standard error of duplicates.

**Supplementary Figure 3: Western blots of serum and purified clusterin.** Purified clusterin (80 ng/lane, and normal human serum (NHS; 1 µl/lane) were separated on SDS PAGE under NR and R conditions (4-12% gel), blotted to nitrocellulose, and detected with clusterin mAbs (2 µg/ml) or secondary (2°) only control. Data for mAbs 2D5 and 4C7 is shown in Figure 1b. 6C12 and 6D1 specifically recognised denatured clusterin under NR and R conditions; 1A4, 1A5, 3C9, 5D11 displayed weak recognition of denatured clusterin and strong non-specific binding to a ~150 kDa protein under NR conditions, potentially human IgG.

**Supplementary Figure 4: Cross-reactivity with rodent clusterin.** (**a**) Titration of human, mouse, and rat serum (4–0.002%) in the in-house clusterin sandwich ELISA; the assay does not detect rodent clusterin. (**b**) Uncropped western blots of normal mouse serum (NMS) and normal rat serum (NRS) displaying detection of clusterin (red arrow) with biotinylated 2D5 and 4C7 (both at 2 µg/ml) under non-reducing (NR) and reducing (R) conditions (1 µl serum/lane, 4-12% gel), including streptavidin-HRP secondary (2°) only control. 2D5 detected mouse and rat clusterin under NR conditions, 4C7 displayed poor cross-reactivity.

**Supplementary Figure 5: Uncropped brain western blots.** (**a**) Uncropped western blots of control TBH and synaptoneurosome (SN) samples displaying detection of clusterin under reducing conditions (10 µg/lane, 3-8% gel), including streptavidin-HRP secondary (2°) only control. (**b**) Uncropped ponceau stain displaying total protein transferred onto the membrane that was probed with anti-clusterin mAbs. Western blot methods are found in supplementary methods.

**Supplementary Figure 6:** **Sex-disaggregated astrocyte and synapse immunostaining data.** (**a**) Clusterin (4C7) colocalisation with GFAP-positive astrocytes in controls (Ctrl) and AD cases from Figure 6c, disaggregated by sex; males and females were separately analysed by unpaired *t-*test and Welch’s *t*-test, respectively, with no significant differences observed (p>0.05). (**b**) Quantification of GFAP/clusterin-positive astrocytes proximal (<20 µm) and distal (>20 µm) to plaques in individual AD samples from Figure 6d, linked by dotted lines and disaggregated by sex. Males and females were separately analysed by Wilcoxon test and paired *t*-test, respectively; differences between plaque-proximal and distal astrocytes remained significant in males (p=0.0312) but fell short of significance in females (p=0.0562), likely due to disaggregation reducing statistical power. (**c**) Quantification of the percentage of mature synapses (puncta positive for both Bassoon and Homer1) positive for clusterin (4C7) in controls and AD cases from Figure 7c, disaggregated by sex; males and females were separately analysed via unpaired *t*-tests with no significant differences observed (p>0.05). (**d**) Quantification of the percentage of total synapses (Bassoon-positive puncta) co-localised with clusterin (4C7) in controls and AD cases from Figure 7d, disaggregated by sex; males and females were separately analysed via unpaired *t*-tests with no significant differences observed (p>0.05). Error bars in all plates represent standard error.

## Supplementary Tables

| **ID** | **Diagnosis** | **Sex** | **Age (years)** | **Tissue area** |
| --- | --- | --- | --- | --- |
| SD037/18 | AD | Male | 72 | Superior temporal gyrus BA41/42 |
| SD012/17 | Control | Female | 71 | Superior temporal gyrus BA41/42 |
| SD024/17 | Control | Male | 72 | Superior temporal gyrus BA41/42 |
| SD030/18 | Control | Male | 63 | Superior temporal gyrus BA41/42 |
| SD042/18 | Control | Female | 73 | Superior temporal gyrus BA41/42 |
| SD046/17 | Control | Female | 65 | Superior temporal gyrus BA41/42 |

**Supplementary Table 1: Brain tissue from Edinburgh Brain Bank.** BA41/42 is the primary auditory cortex.

| **ID** | **Diagnosis** | **Braak score** | **Sex** | **Age (years)** | **PM delay (hours)** | ***APOE* status** | **Tissue area** |
| --- | --- | --- | --- | --- | --- | --- | --- |
| 20174910 | AD | VI | F | 84 | 79 | 3/4 | Hippocampus |
| 20174915 | AD | VI | M | 75 | 76 | 3/3 | Hippocampus |
| 20174933 | AD | VI | M | 85 | 41 | 3/4 | Hippocampus |
| 20174947 | AD | VI | M | 74 | 24 | 3/4 | Hippocampus |
| 20174950 | AD | VI | M | 82 | 81 | 3/4 | Hippocampus |
| 20174963 | AD | VI | F | 83 | 38 | 3/3 | Hippocampus |
| 20174964 | AD | VI | F | 89 | 52 | 3/4 | Hippocampus |
| 20174965 | AD | VI | M | 95 | 29 | 3/3 | Hippocampus |
| 20174966 | AD | VI | M | 79 | 62 | 4/4 | Hippocampus |
| 20229944 | AD | VI | M | 79 | 28 | 3/3 | Hippocampus |
| 20111016 | Control | I – II | F | 74 | 49 | 2/2 | Hippocampus |
| 20140496 | Control | III – IV | F | 80 | 31 | 3/3 | Hippocampus |
| 20140523 | Control | I – II | M | 85 | 85 | n/a | Hippocampus |
| 20141916 | Control | I – II | F | 81 | 65 | 3/3 | Hippocampus |
| 20150535 | Control | I – II | M | 71 | 25 | n/a | Hippocampus |
| 20152907 | Control | III – IV | M | 94 | 25 | 3/4 | Hippocampus |
| 20152910 | Control | III – IV | M | 91 | 24 | 3/4 | Hippocampus |
| 20160443 | Control | I – II | M | 81 | 26 | 2/4 | Hippocampus |
| 20163903 | Control | I – II | F | 71 | 72 | 3/3 | Hippocampus |
| 20174925 | Control | I – II | M | 82 | 73 | 3/3 | Hippocampus |

**Supplementary Table 2: Brain tissue from Newcastle Brain Tissue Resource.** PM is post-mortem.

| **Gene** | **Forward primer (5’-3’)** | **Reverse primer (5’-3’)** |
| --- | --- | --- |
| *CLU* (Total) | CTATCTGCGGGTCACCAC | CTCAGTGACACCGGAAGGAAC |
| *CLU* (Secreted) | ACAGGGTGCCGCTGACC | CAGCAGAGTCTTCATCATGCC |
| *CLU* (Intracellular) | GGGTGCCGCTGACCGAAAT | GAGTCTTTATCTGTTTCACCCCG |
| *ACTB* | ACCATGGATGATGATATCGC | TCATTGTAGAAGGTGTGGTG |
| *GAPDH* | TGCACCACCAACTGCTTAGC | GGCATGGACTGTGGTCATGAG |

**Supplementary Table 3: Primers.** Designed in-house and purchased from Thermo Fisher Scientific. Total *CLU* mRNA was measured using primers spanning exons 7-8, secreted *CLU* mRNA was measured using primers spanning exons 1-2, and intracellular *CLU* mRNA was measured using primers spanning exons 1-3 excluding exon 2.

## Supplementary Methods

### Animals

All animal procedures performed were compliant with the Animals Scientific Procedures Act 1986 and local institutional regulations. C57BL/6 mice (Envigo) were group-housed in environmentally enriched cages, under standard pathogen-free conditions, with a 12-hour light/dark cycle, and *ad libitum* access to food and water. Mice were humanely sacrificed via asphyxiation with increasing CO_2_ concentration; death was confirmed by palpation.

### Direct ELISA

To screen for specific anti-clusterin antibodies, immunosorbent 96-well plates were coated with pure clusterin at 0.5 µg/ml (50 µl/well) in carbonate buffer for 1 hour at 37°C. Plates were blocked with PBS-T with 2% (w/v) BSA for 30 min at 37°C, washed with PBS-T, hybridoma supernatants (50 µl/well) added and incubated at 37°C for 1 hour. Plates were washed, incubated with HRP-conjugated donkey anti-mouse IgG (50 µl/well; 1:5000 in PBS-T; Jackson ImmunoResearch, #715-035-151) for 1 hour at 37°C, washed, and developed with OPD. Absorbance was measured as before and clones with the strongest signal were selected for recloning.

### SDS-PAGE

Protein samples were prepared by diluting with 5x loading dye (250 mM Tris-HCl, pH 6.8; 50% (v/v) glycerol, 5% (w/v) SDS, 0.1% (w/v) bromophenol blue) with or without 5% (v/v) β-mercaptoethanol, then heating at 95°C for 10 mins. PageRuler Plus (Thermo Fisher Scientific, #26620) or HiMark (Thermo Fisher Scientific, #LC5699) pre-stained protein ladders were used as molecular weight markers. Samples were resolved on NuPAGE 4-12% Bis-Tris gels (Invitrogen), NuPAGE 3–8 % Tris-acetate gels (Invitrogen), or 12.5% Bis-Tris polyacrylamide gels (in-house). Protein staining was performed using InstantBlue Coomassie Protein Stain (Abcam, #ab119211) and imaged via autoexposure (G:BOX Chemi XX6, Syngene).

For western blotting, gels were transferred onto Amersham Protran 0.45 Nitrocellulose membrane (Cytiva). Membranes were then stained with Ponceau S (Thermo Fisher Scientific), imaged as above, and quantified by densitometry using ImageJ (National Institutes of Health). Membranes were blocked in PBS-T with 5% (w/v) BSA (blocking buffer) for 1 hour at RT, followed by incubation with anti-clusterin mAbs (2 µg/ml in blocking buffer) overnight at 4°C. Membranes were washed three times with PBS-T, incubated with HRP-conjugated donkey anti-mouse IgG (1:5,000 in blocking buffer) or streptavidin-HRP (1:200 in blocking buffer) for 1 hour at RT, washed as above, then developed with Amersham enhanced chemiluminescence reagent (Cytiva, #GERPN2106) and imaged as before.

### RT-qPCR

RNA was extracted from terminally differentiated iPSC-derived astrocytes using the RNeasy Plus Mini Kit (Qiagen, #74134) with DNase I (Qiagen; #79254). RNA purity and concentration were determined using a DeNovix DS-11 spectrophotometer and cDNA synthesis was performed with a High-Capacity RNA-to-cDNA Kit (Thermo Fisher Scientific, #4387406). RT-qPCR was performed in triplicate in 10 µl reaction volumes with PowerUp SYBR Green Master Mix (Applied Biosystems, #A25742), 0.5 µM forward and reverse primers (Supplementary Table 3), 0.5 µl cDNA, and nuclease-free H_2_O with the following protocol: 2 min at 50°C, 10 min at 95°C; 40 cycles comprising 15 seconds at 95°C, 1 min at 60°C, 15 seconds at 95°C. The ΔCt method was used to normalise gene expression to housekeeping controls *GAPDH* and *ACTB*.
